# Recombinant Goose Circoviruses Circulating in Domesticated and Wild Geese in Poland

**DOI:** 10.3390/v10030107

**Published:** 2018-03-02

**Authors:** Tomasz Stenzel, Daria Dziewulska, Brejnev M. Muhire, Penelope Hartnady, Simona Kraberger, Darren P. Martin, Arvind Varsani

**Affiliations:** 1 Department of Poultry Diseases, Faculty of Veterinary Medicine, University of Warmia and Mazury, 10-719 Olsztyn, Poland; daria.pestka@uwm.edu.pl; 2 Computational Biology Group, Institute of Infectious Disease and Molecular Medicine, Faculty of Health Sciences, University of Cape Town, Observatory, Cape Town 7925, South Africa; mubrejnev@gmail.com (B.M.M.); penelope22hart@gmail.com (P.H.); darrenpatrickmartin@gmail.com (D.P.M.); 3 Department of Molecular Biology, Massachusetts General Hospital, Harvard Medical School, Boston, MA 02114, USA; 4 The Biodesign Center for Fundamental and Applied Microbiomics, Center for Evolution and Medicine, School of Life sciences, Arizona State University, Tempe, AZ 85287, USA; simona.kraberger@asu.edu; 5 Structural Biology Research Unit, Department of Integrative Biomedical Sciences, University of Cape Town, Rondebosch, Cape Town 7701, South Africa

**Keywords:** circovirus, goose, diversity, recombination, ssDNA virus, secondary structure, selection

## Abstract

Circoviruses are circular single-stranded DNA (ssDNA) viruses that infect a variety of animals, both domestic and wild. Circovirus infection in birds is associated with immunosuppression and this in turn predisposes the infected animals to secondary infections that can lead to mortality. Farmed geese (*Anser anser*) in many parts of the world are infected with circoviruses. The majority of the current genomic information for goose circoviruses (GoCVs) (*n* = 40) are from birds sampled in China and Taiwan, and only two genome sequences are available from Europe (Germany and Poland). In this study, we sampled 23 wild and 19 domestic geese from the Gopło Lake area in Poland. We determined the genomes of GoCV from 21 geese; 14 domestic Greylag geese (*Anser anser)*, three wild Greylag geese (*A. anser*), three bean geese (*A. fabalis*), and one white fronted goose (*A. albifrons*). These genomes share 83–95% nucleotide pairwise identities with previously identified GoCV genomes, most are recombinants with exchanged fragment sizes up to 50% of the genome. Higher diversity levels can be seen within the genomes from domestic geese compared with those from wild geese. In the GoCV capsid protein (*cp*) and replication associated protein (*rep*) gene sequences we found that episodic positive selection appears to largely mirror those of beak and feather disease virus and pigeon circovirus. Analysis of the secondary structure of the ssDNA genome revealed a conserved stem-loop structure with the G-C rich stem having a high degree of negative selection on these nucleotides.

## 1. Introduction

Circoviruses (genus: *Circovirus*; family: *Circoviridae*) are non-enveloped, icosahedral viruses, with circular single-stranded DNA (ssDNA) genomes which are approximately 2 kb in length. Transcription is bidirectional with a replication-associated protein (Rep) encoded on the virion sense strand and a capsid protein (CP) on the complementary sense strand [1,2]. Circoviruses are known to infect various bird, mammal, and fish species [1]. The main consequence of circovirus associated infections in birds is immunosuppression which is associated with the apoptosis of lymphocytes [3,4,5,6]. This immunosuppression can predispose circovirus infected birds to secondary infections by pathogenic bacteria, fungi, and viruses [7].

Goose circovirus (GoCV) was firstly described by Soike*,* et al. [8] in a German commercial geese flock showing runting syndrome that was associated with a high degree of mortality. Subsequently, GoCV has been found infecting domesticated geese in other European and Asian countries [9,10,11,12,13] and, more recently, the virus has also been found in wild geese [14]. Based on published data, the prevalence of GoCV infections in domesticated geese (~20–56%) is similar to that noted for circovirus infections in pet/wild parrots (~20–64%) but generally lower than that for circoviruses infecting pigeons (~36–100% depending on age and health status) [9,12,15,16,17,18].

Geese infected with GoCV often shows non-specific clinical symptoms such as diarrhea and growth retardation [5]. Feather disorders, similar to those observed in circovirus-infected parrots [19], have also been observed in geese infected with GoCV [5]. Post-mortem examinations of infected birds frequently reveal both splenomegaly and enlargement of the thymus and liver while hemorrhages in the epi- and endocardium, the lungs, and the thymus have also been noted [5]. The most common histopathological changes are depletion of T-lymphocytes in lymphoid organs including the thymus, the spleen, and the bursa of Fabricius [5]. There have also been cases where GoCV has been found in co-infections with West Nile virus [11,12].

Although 42 full GoCV genomes are presently available in GenBank, all but one sequence from a domesticated goose in Germany and another from a wild graylag goose in Poland, have been obtained from two countries; China (*n* = 15) and Taiwan (*n* = 25). With the intention of determining the diversity of GoCV in Europe we screened wild and domestic geese in Poland for GoCV infections and, in birds when infections were detected we cloned and sequenced full GoCV genomes.

## 2. Materials and Methods

### 2.1. Sample Collection and Processing

Spleen and liver sections were collected during necropsy from 23 wild geese and one mallard duck that had been shot by sport hunters in the area around Gopło Lake in Kujawsko-Pomorskie District in Poland. Similar tissue types were also sampled from slaughtered domestic geese (*n* = 19) from farms located in the areas around Gopło Lake. Sample descriptions are provided in Table 1. Forty four tissue samples (each approximately 50 mg) were individually homogenized in 500 µL PBS using Tissuelyser II (Qiagen, Hilden, Germany). The supernatant from the homogenate (~200 µL) was used for DNA isolation using a Janus automated workstation (Perkin Elmer, Waltham, MA, USA) and NucleoMag Tissue Kit (Macherey-Nagel, Düren, Germany).

### 2.2. Screening and GoCV Genome Recovery

Each sample was prescreened with the broad-spectrum nested PCR method targeting the *rep* genes of various avian circoviruses in accordance with Halami et al. [20]. For each positive sample, circular DNA was amplified using 1 µL of total DNA and TempliPhi (GE Healthcare, Marlborough, MA, USA) as previously described [16,21]. Full GoCV genomes were recovered using the enriched DNA as a template with back-to-back primer pair GoCV-F 5′-CTSTCTCGWGCYCGGGGATCTGAC-3′ and GoCV-R 5′-CCAGGCTCTTCCTCCCAGCKWCTCTT-3′ using Kapa HiFI Hotstart DNA polymerase (Kapa Biosystems, Wilmington, DE, USA) with the following thermal cycling protocol: 96 °C for 3 min, 25 cycles (98 °C (20 s), 60 °C (30 s), 72 °C (2 min)), 72 °C for 3 min. Amplicons were resolved on 0.7% agarose gels and ~2 kb fragments were excised. Gel excised fragments were purified using a MEGA-spin Agarose Gel DNA Extraction Kit (iNtRON Biotechnology, Daejeon, Korea). Cleaned products were ligated into the plasmid pJET 2.1 (Thermo Fisher Scientific, Waltham, MA, USA) and transformed into DH-5α *Escherichia coli* competent cells. Recombinant plasmid DNA was isolated from single *E. coli* colonies using a DNA-spin Plasmid DNA Extraction Kit (iNtRON Biotechnology, Daejeon, Korea) and these were subsequently Sanger sequenced (Macrogen Inc., Seoul, Korea). Full genome coverage was obtained using primer walking. Sequence contigs were assembled into full genomes using DNA Baser V4 (Heracle BioSoft S.R.L., Pitesti, Romania).

### 2.3. Bioinformatic Analysis

The genetic diversity among the GoCV isolates was analyzed using SDT v1.2 [22]. Given that recombination has been detected in a range of other circoviruses, prior to phylogenetic analysis the 63 GoCV full genome sequences were aligned using MUSCLE [23] and analyzed for recombination using the seven detection methods implemented in RDP version 4.70 [24]. A maximum likelihood (ML) phylogenetic tree accounting for recombination was then constructed with PHYML 3.0 [25] using the full genome sequence alignment from which all recombinationally inherited genome segments had been removed. This tree was inferred with the best-fit substitution model GTR + G determined using jModelTest [26], with 1000 bootstrap replicates used to infer branch support and rooted with two swan circovirus sequences (EU056309, EU056310). Branches with less than 60% support were collapsed using TreeGraph2 [27].

The GoCV, PiCV, and BFDV gene sequences were all initially codon aligned within a singled data set so as to ensure that homologous codon sites could be accurately compared to one another following selection analyses. Gene sequences from each of the three species were then analyzed separately to identify codon sites evolving under either positive or negative selection using FUBAR [28] and under episodic positive selection using MEME [29]. We subsequently used the difference between the non-synonymous (dN) and synonymous (dS) rates (dN-dS) obtained from FUBAR and the locations of sites evolving under episodic selection from MEME to generate a selection map comparing the types and degrees of selection between the three circovirus datasets (GoCV; pigeon circovirus, PiCV; beak and feather disease viruses, BFDV) using the computer program SelectionMap (http://www.cbio.uct.ac.za/~brejnev/ComputationalTools.html).

Given that evidence of pervasive biologically relevant secondary structural elements has been found in other circovirus genomes, we used the computer program NASP [30] as previously described by Muhire*,* et al. [31] to identify and rank, in order of conservation, the secondary structural elements that are most likely present within the 63 GoCV genomes. Briefly, this was achieved using the minimum free-energy (MFE) approach implemented in the hybrid-ssmin component of UNAFold (with sequences treated as circular and folding carried out at 37 °C under 0.1 M magnesium and 1 M sodium ionic conditions) [32] to infer ensembles of secondary structural elements within ten GoCV genomes representing the known breadth of GoCV diversity (DQ192281, DQ192285, KP203866, KP229371, KT808650, KT808653, KT808656, KT808657, KT808663, KT808668) and ranking of structures based on the relative degrees to which inferred base-pairing interactions were conserved within them. From these ranked lists of plausible conserved structural elements, subsets of high-confidence structural elements—referred to as a high-confidence structure set (HCSS)—were identified using a nucleotide-shuffling permutation test (with 100 permutations and a *p*-value cutoff 0.05). In subsequent analyses, the only nucleotides considered as being paired within secondary structures were those identified by NASP as being base-paired within the HCSSs, while all other nucleotides were treated as unpaired sites. Structures were visualized with overlaid evolutionary data using the computer program DOOSS [33] and compared to similar structures in other circovirus genomes (namely beak and feather disease viruses (BFDV) and pigeon circovirus (PiCV), which were analyzed previously using identical methods [21].

## 3. Results and Discussion

### 3.1. GoCV in Domestic and Wild Geese in Poland

We recovered the genomes of GoCV from 14 domestic Greylag geese (*Anser anser)* and three wild Greylag geese (*A. anser*), three bean geese (*Anser fabalis*), and one white fronted goose (*Anser albifrons*) (Table 2; GenBank accession #s KT808650–KT808670). The 21 genomes share between 83% and 95% pairwise identities with other GoCV genomes available in GenBank (Figure S1). One of the GoCV genome sequences (GenBank accession # KT808657; Figure S1) from a domestic Greylag goose was most closely related to a divergent GoCV sequence recovered from a Polish wild Greylag goose (89%) [14] and, collectively, these two genomes share <84% genome-wide pairwise identity with all other known GoCV genomes.

**Figure 1:**
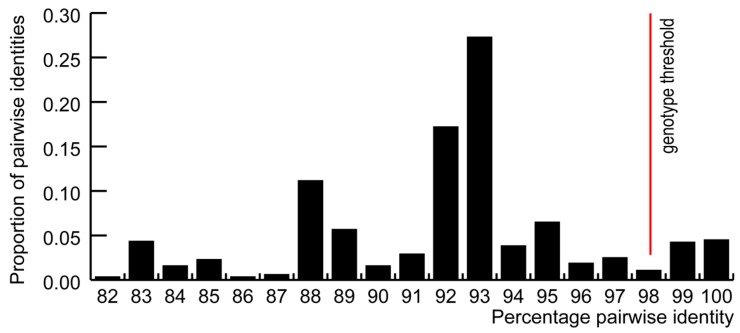
Distribution of pairwise identities of the 63 GoCV genomes. For the purpose of this study, a 98% pairwise identity was used as genotype threshold.

**Figure 2:**
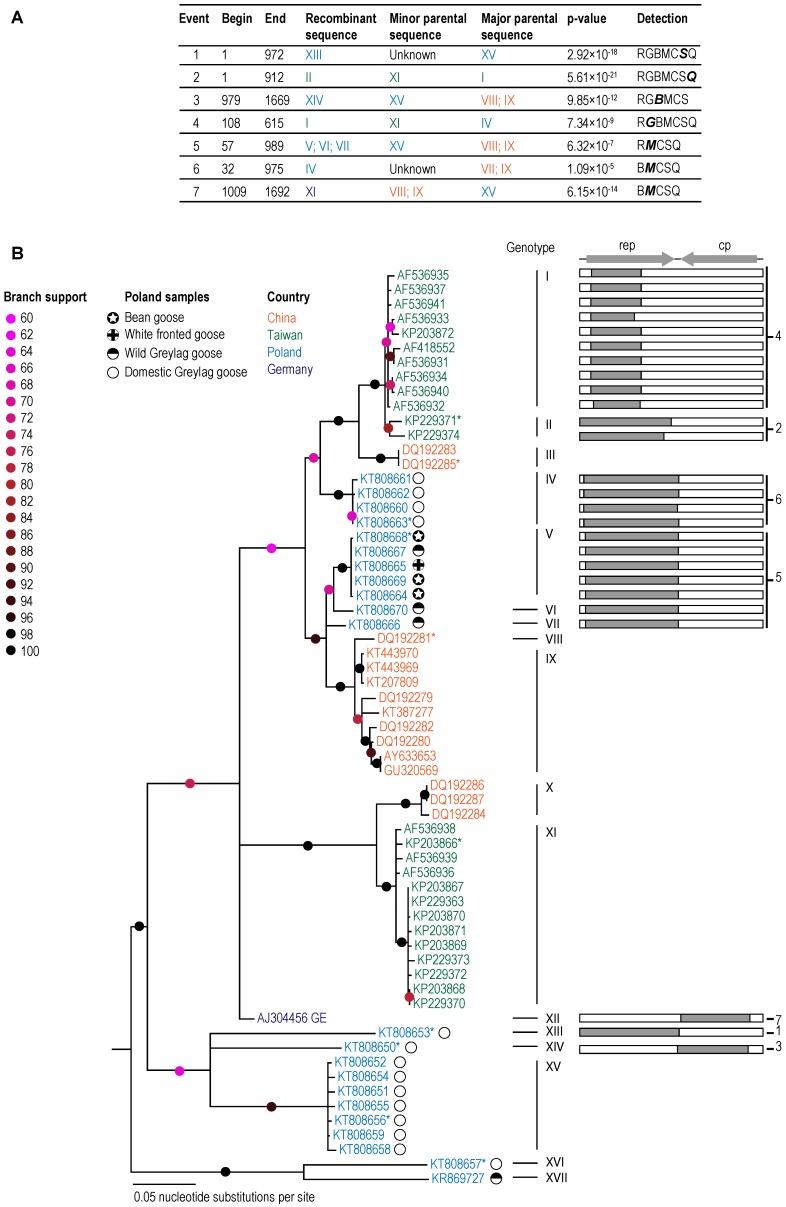
(**A**) A summary of the seven recombination events detected using the RDP (R), GENECONV (G), BOOTSCAN (B), MAXCHI (M), CHIMAERA (C), SISCAN (S), and 3SEQ (Q) methods implemented in the computer program RDP4. Only detection methods with associated *p*-values < 0.05 are shown. The *p*-value is for the detection method shown in bold italics. (**B**) Recombination free Maximum likelihood phylogenetic tree (left) with a cartoon illustration of the associated recombination event. Genome sequences used in DNA secondary structure analysis are marked with *.

In order to rationally categorize the presently known GoCV sequences, we determined the distribution of genome-wide pairwise identities (Figure 1). Owing to the trough in this distribution at 98% identity (indicating that there are very few sequences that share 98% genome-wide pairwise identity), we opted to use 98% as a threshold for assigning the genomes to different genotype groupings. According to this threshold, the 63 known GoCV genomes can be assigned to 17 genotypes (which we have simply named I through XVII; Figure 2; Table 2). Whereas the Polish sequences fell within nine of these genotype groupings (IV, V, VI, VII, XII, XIII, XIV, XVI, and XVII; Figure 2), the 15 GoCV genomes from China belong to four genotypes (III, VIII, IX, and X), the 25 from Taiwan to three genotypes (I, II, and XI), and the isolate from Germany to genotype XII (Figure 2).

It is noteworthy that the GoCV genotype assignments for the Polish isolates display a degree of host-structure (Figure 2; Table 2). Specifically, the isolates from wild geese belong mainly to the genotypes V, VI, and VII with a divergent Greylag goose isolate [14] falling within genotype XVII. Interestingly, the isolates obtained from the migratory bean and white fronted geese which do not nest in Poland, fall exclusively within genotype V. Conversely the genotype VI, VII, and XVII isolates were from wild Greylag geese which nest in the area of Gopło Lake. The genome-wide pairwise identities that are shared by GoCV isolates from wild migratory geese and those from local population geese are ~97%, whereas the isolates obtained from wild geese belonging to genotype V all share close to 100% pairwise identity with one another.

The GoCV isolates from wild geese were closely related to genotype IV isolates from domestic Greylag geese (94–95% identity). GoCV isolates assigned to genotypes XIII through XV are from domestic Greylag geese used for reproduction which share between 84% and 90% genome-wide pairwise identity with isolates from wild and domestic geese that have been slaughtered. The GoCV genotypes circulating in wild geese in the area of Gopło Lake therefore differ from those found in domestic geese kept in this area.

Seven unique recombination events were identified within 28 of the 63 GoCV sequences with a transferred fragment size spanning between 27% and 50% of the genome (Figure 2). Six of the nine polish genotypes were identified to be recombinant (IV, V, VI, VII, XIII, and XIV; Figure 1). As was suggested by the pairwise sequence analyses, the maximum likelihood phylogenetic tree that was constructed following the removal of recombinationally acquired genome segments displayed strong evidence of geographical structure.

**Figure 3:**
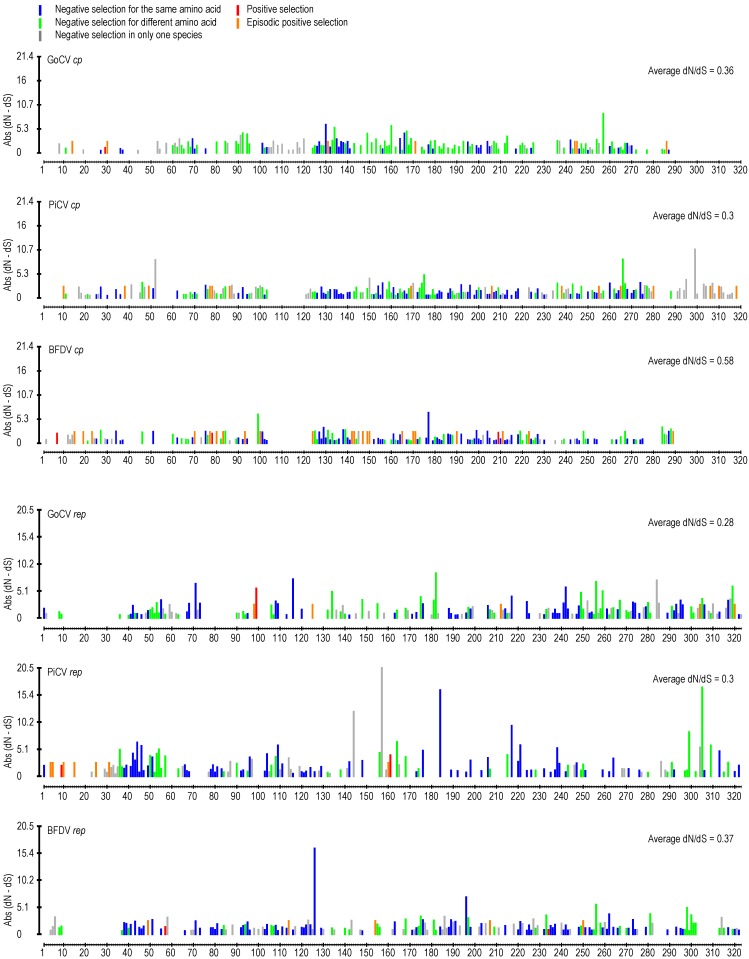
Patterns of natural selection acting at *cp* and *rep* codon sites in beak and feather disease viruses (BFDV), GoCV, and pigeon circovirus (PiCV) genomes. Presented here are schematic representations of aligned *rep* and *cp* codon sites where, for each site, absolute (Abs) values of inferred synonymous substitution rates subtracted from inferred non-synonymous substitution rates (dN-dS) are plotted (as determined by the FUBAR method). Significantly positive dN-dS values are indicated by a red bar (indicating the strength of positive selection), and significantly negative dN-dS values are plotted in grey, green, and blue (indicating the strength of negative selection). Whereas blue colors indicate sites at which negative selection favors the same encoded amino acid in multiple different species, green colors indicate sites where negative selection favors different encoded amino acids in different species. Grey colors indicate sites at which negative selection was only detectable in a single species. Sites in orange display evidence of evolving under positive selection within particular lineages of the various species (indicated by the MEME method). Missing bars indicates gaps in the BFDV, GoCV, and PiCV gene alignment.

We detected and compared natural selection signals within codon alignments of the GoCV *rep* and *cp* genes using two different codon-by-codon selection detection approaches (Figure 3; Supplementary data 1). In the BFDV *cp* there are three instances of positive selection, as opposed to none in PiCV and one in GoCV. The instances of episodic positive selection appears to largely mirror each other. The negative selection appears to be higher in the *rep* genes compared to *cp* (based on individual dN/dS values).

### 3.2. DNA Secondary Structure Analysis of GoCV Genomes

**Figure 4:**
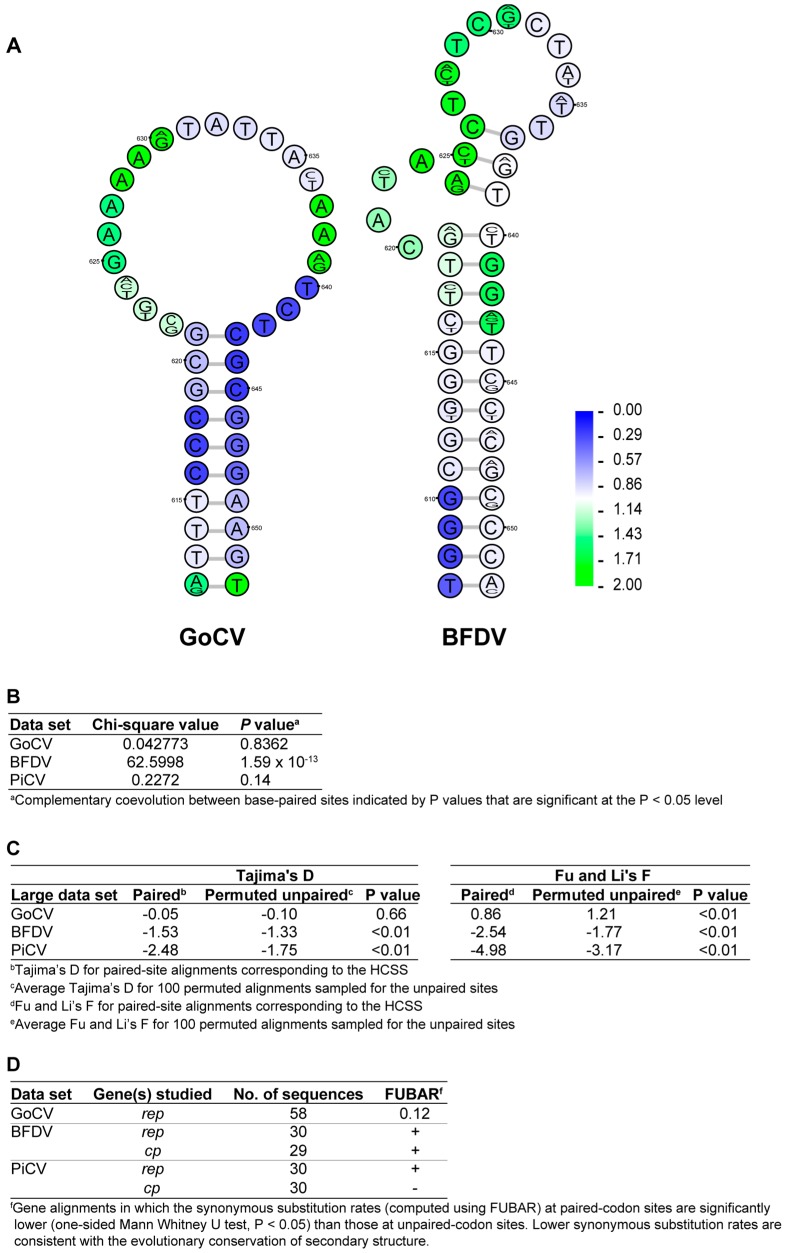
(**A**) Conserved stem-loop structures within *rep* of GoCV and BFDV genomes. The structures presented are the consensus of all available genomes and were amongst the most highly conserved of all plausible structural elements detected within the genomes of these two species (2nd out of 137 in GoCV and 9th out of 143 in BFDV). The rank ratio indicates the actual conservation rank of the structure over the total number of predicted secondary structures. Nucleotide sequence variability is reflected by a sequence logo at each position, while overlaid synonymous substitution rate estimates are represented by the shading of each nucleotide (ranging from blue for low to green for high). Although these two structures have no obvious sequence similarity, something expected given that GoCV and BFDV groups are very divergent (sharing 58.5% sequence identity), they form within same genomic region in *rep* and have similar conformation consisting of a stable stem-loop structure with a GC-rich stem-region and with evidence of low synonymous substitution rates in codons occurring within the stem-region consistent with strong selection acting against synonymous substitutions at these sites. (**B**) Association between paired sites and complementarily coevolving sites. (**C**) Tajima’s D and Fu and Li F statistics for paired and unpaired genomic site alignments. (**D**) Comparison of synonymous substitution rates at paired- and unpaired-codon sites.

The stem-loop structures in GoCV and BFDV visualized in Figure 4A were both highly conserved among the plausible structural elements detected within the genomes of these two species (2nd out of 137 in GoCV and 9th out of 143 in BFDV). Despite sharing no obvious sequence similarity, the similar GC-rich, stable stem-loop conformation and genomic location of the structural elements in *rep* could indicate shared biological function across viral species. The excessively low synonymous substitution rates observable in the stem region of both structures (indicated by the blue coloring of the nucleotides) indicates a high degree of negative selection acting at the nucleotide-level for these particular sites.

Tests designed to determine whether the evolution of GoCV sequences was consistent with the selective preservation of biologically functional structural elements within their genomes were applied exactly as described by Muhire et al. [31]. These tests compared paired sites within the HCSS structures to unpaired sites with respect to degrees of evolutionary neutrality, synonymous substitution rate, and rates of complementary coevolution. Identical evolutionary analyses to those performed here on GoCV have been previously carried out on BFDV and PiCV [21], enabling the direct comparison of these three circovirus species.

At the whole-genome-scale, neutrality tests (which compare frequencies of minor/alternative allele frequencies at polymorphic paired sites with those occurring at unpaired sites) were used to test for elevated degrees of purifying selection at paired sites relative to unpaired sites. For all 3 datasets examined (GoCV, BFDV, and PiCV), minor allele frequencies were significantly lower at paired sites than at unpaired sites as indicated by lower D and F test static values (*p* < 0.01 in all cases), with the exception of GoCV (*p* = 0.66, Tajima’s D test) (Figure 4C). This finding is consistent with purifying selection being stronger at paired-nucleotide sites within the HCSS than at the remainder of unpaired genomic sites. This strong signal of purifying selection indicates that a substantial proportion of paired sites within the HCSS of the analyzed genomes are evolving in a manner that is consistent within many of the parent structures being evolutionarily preserved.

A very strong association between nucleotide sites that are complementary coevolving and nucleotide sites that are base-paired was detected in the BFDV dataset (*p* = 1.59 × 10^−13^) (Figure 4B). This provides compelling evidence that at least a subset of the structures in the BFDV genome likely provide a significant fitness advantage to the viruses, which suggests that these structures may have crucial, as yet undetermined biological functions. The lack of significant evidence of coevolution between paired-nucleotides within the GoCV HCSS may be due to the aforementioned high inter-cluster divergence of the GoCV species, or a lack of detectable biologically important structures.

## 4. Conclusions

The GoCV genome diversity in domesticated geese was found to be higher than that in wild geese. The domestic geese sampled in this study were slaughter birds which were from parental flocks (reproductive birds). Furthermore, a large number of these genomes are recombinant and this is likely due to the high density of birds in slaughter flocks, and in the herds of wild geese during migration, which facilitate recombination among GoCVs, similar to what has been noted for BFDVs in parrot breeding facilities [16]. The farming of geese in areas that are also inhabited by wild geese may enable the transfer of pathogens between domestic and wild geese populations. Although the GoCV variants from wild and domestic geese are in different genotype groupings, the detection of recombination between viruses assigned to the “wild” and “domestic” genotypes and suggests that may be at least a low degree of GoCV circulation between domestic and wild geese population—possibly even in the area of Gopło Lake. If there are indeed persistent low-levels of viral transmission between wild and domestic birds, this may expose both the domestic birds to pathogens from throughout the seasonal geographical ranges of the wild-birds (which include taiga areas of Scandinavia and that of Central to North Siberia, and the Siberian tundra), and the wild birds throughout those same geographical ranges to pathogens originating within the Polish domestic goose farming sector.

Taking into consideration the high diversity of GoCVs, which is typical for all avian circoviruses, and the fact that these viruses are highly recombinant and evolving at a significantly rapid rate [16,34], full genome analysis allows for the determination viral dynamics amongst local and migratory population of birds.

## Figures and Tables

**Table 1:** Summary of all geese samples included in this study. Samples were prescreened by broad spectrum nested PCR by Halami*,* et al. [20].

Sample ID	Sampling Date	Species	Common Name	Age	Health Status	Broad Spectrum Primer Positive	GoCV Positive	GenBank Accession #
G1	21 November 2014	*Anser fabalis*	Bean goose	A	H	Yes	Yes	KT808664
G2	3 December 2014	*Anser fabalis*	Bean goose	A	H	No	No	N/A
G3	3 December 2014	*Anser fabalis*	Bean goose	A	H	Yes	No	N/A
G4	3 December 2014	*Anser albifrons*	White fronted goose	Y	H	Yes	Yes	KT808665
G5	20 November 2014	*Anser anser*	Wild Greylag goose	A	H	Yes	No	N/A
G6	20 November 2014	*Anser anser*	Wild Greylag goose	Y	H	Yes	Yes	KT808666
G7	20 November 2014	*Anser anser*	Wild Greylag goose	A	H	No	No	N/A
G8	20 November 2014	*Anser anser*	Wild Greylag goose	A	H	Yes	No	N/A
G9	20 November 2014	*Anser anser*	Wild Greylag goose	A	H	Yes	Yes	KT808667
G10	20 November 2014	*Anser albifrons*	White fronted goose	A	H	No	No	N/A
G11	21 November 2014	*Anser albifrons*	White fronted goose	Y	H	No	No	N/A
G12	21 November 2014	*Anser fabalis*	Bean goose	A	H	Yes	No	N/A
G13	21 November 2014	*Anser fabalis*	Bean goose	A	H	No	No	N/A
G14	21 November 2014	*Anser fabalis*	Bean goose	A	H	No	No	N/A
G15	21 November 2014	*Anser fabalis*	Bean goose	A	H	Yes	Yes	KT808668
G16	21 November 2014	*Anser fabalis*	Bean goose	A	H	Yes	Yes	KT808669
G17	21 November 2014	*Anser fabalis*	Bean goose	A	H	No	No	N/A
G18	22 November 2014	*Anser anser*	Wild Greylag goose	A	H	Yes	No	N/A
G19	22 November 2014	*Anser anser*	Wild Greylag goose	A	H	Yes	Yes	KT808670
G20	22 November 2014	*Anser anser*	Wild Greylag goose	A	H	Yes	No	N/A
G21	23 November 2014	*Anser albifrons*	White fronted goose	A	H	Yes	No	N/A
G22	23 November 2014	*Anser fabalis*	Bean goose	A	H	No	No	N/A
G23	23 November 2014	*Anser fabalis*	Bean goose	A	H	No	No	N/A
G24	23 November 2014	*Anas platyrhynchos*	Mallard duck	A	H	Yes	No	N/A
DG1	6 December 2014	*Anser anser*	Domestic goose	A *	H	Yes	Yes	KT808650
DG2	6 December 2014	*Anser anser*	Domestic goose	A *	H	Yes	No	N/A
DG3	6 December 2014	*Anser anser*	Domestic goose	A *	H	Yes	Yes	KT808651
DG4	6 December 2014	*Anser anser*	Domestic goose	A *	H	Yes	Yes	KT808652
DG5	6 December 2014	*Anser anser*	Domestic goose	A *	H	Yes	Yes	KT808653
DG6	6 December 2014	*Anser anser*	Domestic goose	A *	H	Yes	Yes	KT808654
DG7	6 December 2014	*Anser anser*	Domestic goose	A *	H	Yes	Yes	KT808655
DG8	6 December 2014	*Anser anser*	Domestic goose	A *	H	Yes	Yes	KT808656
DG9	5 December 2014	*Anser anser*	Domestic goose	A *	H	Yes	No	N/A
DG10	5 December 2014	*Anser anser*	Domestic goose	A *	H	Yes	No	N/A
DG11	5 December 2014	*Anser anser*	Domestic goose	A *	H	Yes	Yes	KT808657
DG12	5 December 2014	*Anser anser*	Domestic goose	A *	H	Yes	No	N/A
DG13	5 December 2014	*Anser anser*	Domestic goose	A *	H	Yes	Yes	KT808658
DG14	5 December 2014	*Anser anser*	Domestic goose	A *	H	Yes	Yes	KT808659
DG15	5 June 2015	*Anser anser*	Domestic goose	Y **	S ***	Yes	Yes	KT808660
DG16	5 June 2015	*Anser anser*	Domestic goose	Y **	S ***	Yes	Yes	KT808661
DG17	5 June 2015	*Anser anser*	Domestic goose	Y **	S ***	Yes	Yes	KT808662
DG18	5 June 2015	*Anser anser*	Domestic goose	Y **	S ***	Yes	Yes	KT808663
DG19	5 June 2015	*Anser anser*	Domestic goose	Y **	S ***	Yes	No	N/A

A: Adult; Y: Young; H: Healthy; S: Sick; * Reproductive/parental flock; ** Slaughter flock; *** Aspergillosis.

**Table 2:** Details of 63 Goose circovirus (GoCV) genome sequences used in this study. GoCV sequences determined in this study are in bold font (GenBank accession #s KT808650–KT808670).

Accession	Description	Country	Host	Genotype
AF418552	Goose circovirus isolate TW	Taiwan	*Anser anser*	I
AF536931	Goose circovirus isolate TW1/2001	Taiwan	*Anser anser*	I
AF536932	Goose circovirus isolate TW2/2001	Taiwan	*Anser anser*	I
AF536933	Goose circovirus isolate TW3/2001	Taiwan	*Anser anser*	I
AF536934	Goose circovirus isolate TW4/2001	Taiwan	*Anser anser*	I
AF536935	Goose circovirus isolate TW5/2001	Taiwan	*Anser anser*	I
AF536936	Goose circovirus isolate TW6/2001	Taiwan	*Anser anser*	XI
AF536937	Goose circovirus isolate TW7/2001	Taiwan	*Anser anser*	I
AF536938	Goose circovirus isolate TW8/2001	Taiwan	*Anser anser*	XI
AF536939	Goose circovirus isolate TW9/2001	Taiwan	*Anser anser*	XI
AF536940	Goose circovirus isolate TW10/2001	Taiwan	*Anser anser*	I
AF536941	Goose circovirus isolate TW11/2001	Taiwan	*Anser anser*	I
AJ304456	Goose circovirus isolate DE	Germany	*Anser* sp.	XII
AY633653	Goose circovirus isolate yk1	China	*Anser anser*	IX
DQ192279	Goose circovirus isolate yk2	China: Zhejiang	*Anser anser*	IX
DQ192280	Goose circovirus isolate yk3	China: Zhejiang	*Anser anser*	IX
DQ192281	Goose circovirus isolate yk4	China	*Anser anser*	VIII
DQ192282	Goose circovirus isolate xs1	China	*Anser anser*	IX
DQ192283	Goose circovirus isolate xs2	China	*Anser anser*	III
DQ192284	Goose circovirus isolate xs3	China	*Anser anser*	X
DQ192285	Goose circovirus isolate xs4	China	*Anser anser*	III
DQ192286	Goose circovirus isolate xs5	China	*Anser anser*	X
DQ192287	Goose circovirus isolate xs6	China	*Anser anser*	X
GU320569	Goose circovirus isolate JX1	China: Jiangxi	*Anser anser*	IX
KP203866	Goose circovirus isolate 1020111GB	Taiwan: Yunlin	*Coscoroba coscoroba*	XI
KP203867	Goose circovirus isolate 1021024G	Taiwan: Yunlin	*Coscoroba coscoroba*	XI
KP203868	Goose circovirus isolate GB20-13	Taiwan: Kaohsiung	*Coscoroba coscoroba*	XI
KP203869	Goose circovirus isolate GB21-9	Taiwan: Pingtung	*Coscoroba coscoroba*	XI
KP203870	Goose circovirus isolate GB25-8	Taiwan: Pingtung	*Coscoroba coscoroba*	XI
KP203871	Goose circovirus isolate GB26-15	Taiwan: Changhua	*Coscoroba coscoroba*	XI
KP203872	Goose circovirus isolate GB27-20	Taiwan: Yunlin	*Anser cygnoides*	I
KP229363	Goose circovirus isolate CF13001	Taiwan: Yunlin	*Coscoroba coscoroba*	XI
KP229370	Goose circovirus isolate CD13088	Taiwan: Chiayi	*Coscoroba coscoroba*	XI
KP229371	Goose circovirus isolate CPA13007-1	Taiwan	*Coscoroba coscoroba*	II
KP229372	Goose circovirus isolate CJ14010	Taiwan: Chiayi	*Coscoroba coscoroba*	XI
KP229373	Goose circovirus isolate CPA13007-2	Taiwan	*Coscoroba coscoroba*	XI
KP229374	Goose circovirus isolate CPA14012	Taiwan	*Coscoroba coscoroba*	II
KR869727	Goose circovirus isolate 2GK	Poland	*Anser anser*	XVII
KT207809	Goose circovirus isolate TD254-2014	China	*Anser anser*	IX
KT387277	Goose circovirus isolate Shandong	China	*Anser anser*	IX
KT443969	Goose circovirus isolate TD227/2013	China	*Anser anser*	IX
KT443970	Goose circovirus isolate TD265/2013	China	*Anser anser*	IX
KT808650	Goose circovirus isolate DG1	Poland	*Anser anser*	XIV
KT808651	Goose circovirus isolate DG3	Poland	*Anser anser*	XV
KT808652	Goose circovirus isolate DG4	Poland	*Anser anser*	XV
KT808653	Goose circovirus isolate DG5	Poland	*Anser anser*	XIII
KT808654	Goose circovirus isolate DG6	Poland	*Anser anser*	XV
KT808655	Goose circovirus isolate DG7	Poland	*Anser anser*	XV
KT808656	Goose circovirus isolate DG8	Poland	*Anser anser*	XV
KT808657	Goose circovirus isolate DG11	Poland	*Anser anser*	XVI
KT808658	Goose circovirus isolate DG13	Poland	*Anser anser*	XV
KT808659	Goose circovirus isolate DG14	Poland	*Anser anser*	XV
KT808660	Goose circovirus isolate DG15	Poland	*Anser anser*	IV
KT808661	Goose circovirus isolate DG16	Poland	*Anser anser*	IV
KT808662	Goose circovirus isolate DG17	Poland	*Anser anser*	IV
KT808663	Goose circovirus isolate DG18	Poland	*Anser anser*	IV
KT808664	Goose circovirus isolate G1	Poland	*Anser fabalis*	V
KT808665	Goose circovirus isolate G4	Poland	*Anser albifrons*	V
KT808666	Goose circovirus isolate G6	Poland	*Anser anser*	VII
KT808667	Goose circovirus isolate G9	Poland	*Anser anser*	V
KT808668	Goose circovirus isolate G15	Poland	*Anser fabalis*	V
KT808669	Goose circovirus isolate G16	Poland	*Anser fabalis*	V
KT808670	Goose circovirus isolate G19	Poland	*Anser anser*	VI

## Supplementary Materials

The following are available online at http://www.mdpi.com/1999-4915/10/3/107/s1, Figure S1: Pairwise identity matrix of the 63 GoCV genomes.
