# Supplementary figures and images for: Recombinant Goose Circoviruses Circulating in Domesticated and Wild Geese in Poland
Source: Viruses. 2018 Mar 2;10(3):107. doi: 10.3390/v10030107 (PMC5869500; doi:10.3390/v10030107)

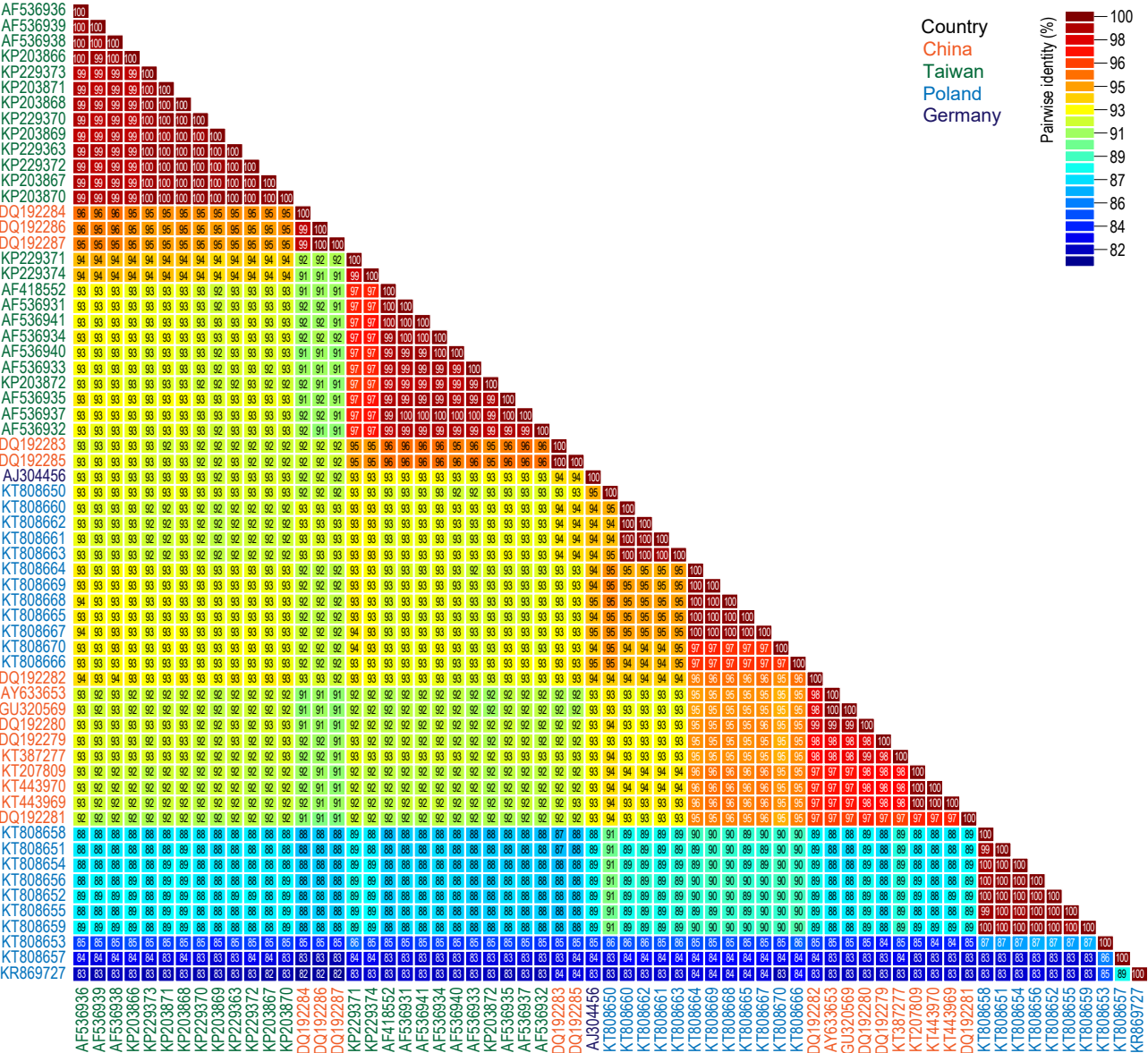

Supplement: Supplementary file 1 [file viruses-10-00107-s001.zip › Supplementary figure 1.pdf]
